# Rheumatoid arthritis increases the risk of heart failure: results from the cross-sectional study in the US population and mendelian randomization analysis in the European population

**DOI:** 10.3389/fimmu.2024.1377432

**Published:** 2024-05-28

**Authors:** Kaisaierjiang Kadier, Diliyaer Dilixiati, Xu Zhang, Huan Li, Lirong Kuang, Jian Huang, Xintian Cai, Tao Ling, Fanqi Kong, Xiaozhu Liu

**Affiliations:** ^1^ Department of Cardiology, First Affiliated Hospital of Xinjiang Medical University, Urumqi, China; ^2^ Department of Urology, First Affiliated Hospital of Xinjiang Medical University, Urumqi, China; ^3^ Center for Reproductive Medicine, Chongqing Health Center for Women and Children, Women and Children’s Hospital of Chongqing Medical University, Chongqing, China; ^4^ Chongqing Reproductive Genetics Institute, Chongqing, China; ^5^ Chongqing College of Electronic Engineering, Chongqing, China; ^6^ Department of Ophthalmology, Wuhan Wuchang Hospital (Wuchang Hospital Affiliated to Wuhan University of Science and Technology), Wuhan, China; ^7^ Graduate School, Guangxi University of Chinese Medicine, Nanning, China; ^8^ Department of Diagnostic Ultrasound, Sir Run Run Shaw Hospital, Zhejiang University College of Medicine, Hangzhou, China; ^9^ Department of Graduate School, Xinjiang Medical University, Urumqi, China; ^10^ Department of Pharmacy, Suqian First Hospital, Suqian, China; ^11^ Department of Cardiology, The Third Affiliated Hospital of Wenzhou Medical University, Wenzhou, China; ^12^ Department of Critical Care Medicine, Beijing Shijitan Hospital, Capital Medical University, Beijing, China

**Keywords:** rheumatoid arthritis, seronegative rheumatoid arthritis, heart failure, national health and nutrition examination survey, mendelian randomization

## Abstract

**Objective:**

Rheumatoid arthritis (RA) is a chronic systemic autoimmune disease. Among its various complications, heart failure (HF) has been recognized as the second leading cause of cardiovascular death in RA patients. The objective of this study was to investigate the relationship between RA and HF using epidemiological and genetic approaches

**Methods:**

The study included 37,736 participants from the 1999-2020 National Health and Nutrition Examination Survey. Associations between RA and HF in the US population were assessed with weighted multivariate logistic regression analysis. A two-sample Mendelian randomization (MR) analysis was employed to establish the causal relationship between the two variables. The primary analysis method utilized was inverse variance weighting (IVW). Additionally, horizontal pleiotropy and heterogeneity were assessed to account for potential confounding factors. In cases where multiple independent datasets were accessible during MR analysis, we combined the findings through a meta-analytical approach.

**Results:**

In observational studies, the prevalence of HF in combination with RA reached 7.11% (95%CI 5.83 to 8.39). RA was positively associated with an increased prevalence of HF in the US population [odds ratio (OR):1.93, 95% confidence interval (CI):1.47-2.54, P < 0.0001]. In a MR analysis utilizing a meta-analytical approach to amalgamate the results of the IVW method, we identified a significant causal link between genetically predicted RA and a heightened risk of HF (OR = 1.083, 95% CI: 1.028-1.141; P = 0.003). However, this association was not deemed significant for seronegative RA (SRA) (OR = 1.028, 95% CI: 0.992-1.065; P = 0.126). These findings were consistent across sensitivity analyses and did not indicate any horizontal pleiotropy.

**Conclusion:**

RA correlates with an elevated prevalence of HF within the US population. Furthermore, genetic evidence derived from European populations underscores a causal link between RA and the risk of HF. However this association was not significant in SRA.

## 1 Introduction

Rheumatoid arthritis (RA) is the most prevalent autoimmune-mediated arthritis and is classified as a systemic inflammatory disease. Its prevalence is on the rise every year, making it a significant global healthcare challenge (1). RA can have long-term adverse consequences for patients, including physical disability and reduced quality of life. Furthermore, the presence of extra-articular manifestations can significantly increase the risk of death (2). In fact, there is a strong association between RA and cardiovascular disease (CVD), and RA has been identified as an independent risk factor for CVD, which contributes to the high mortality rate of RA (3, 4). Systemic inflammation and immune dysfunction serve as pathways that promote and accelerate CVD in patients with RA, ultimately leading to increased morbidity and mortality. It is important to address these underlying factors in order to improve outcomes for these patients (5, 6).

Heart failure (HF) is the second leading cause of cardiovascular mortality in patients with RA, with studies indicating that RA doubles the incidence of HF compared to those without the condition (7–9). Interestingly, the exact mechanism behind this heightened risk is still unclear. HF is a multifaceted clinical syndrome that involves the interaction and coexistence of multiple etiologies. Among these, hypertension and ischemic heart disease are considered to be the primary risk factors. However, despite the increased prevalence of ischemic cardiomyopathy with hypertension in the RA population, this does not seem to impact the heightened risk of HF (10, 11). Compared to the typical symptom profile of the HF population, patients with both RA and HF may not exhibit the usual signs and symptoms and may have preserved ejection fraction, which could indicate a worse prognosis (12). The evidence presented above suggests a possible causal relationship between RA and HF, which could provide an explanation for the observed association.

In observational studies, it can be challenging to eliminate the influence of residual confounding factors, which makes it difficult to draw causal inferences. However, by using Mendelian randomization (MR) analysis, we can overcome this limitation and obtain more reliable results based on available data (13). Our study aimed to investigate the potential link between RA and HF. To achieve this, we conducted a cross-sectional study using the National Health and Nutrition Examination Survey (NHANES) database, which has a national scope. Additionally, we utilized MR to further explore the causal relationship between the two conditions. Our hypothesis was that RA is associated with a higher prevalence of HF and that there is a positive causal effect between the two.

## 2 Materials and methods

### 2.1 Overall research design

**Figure 1:**
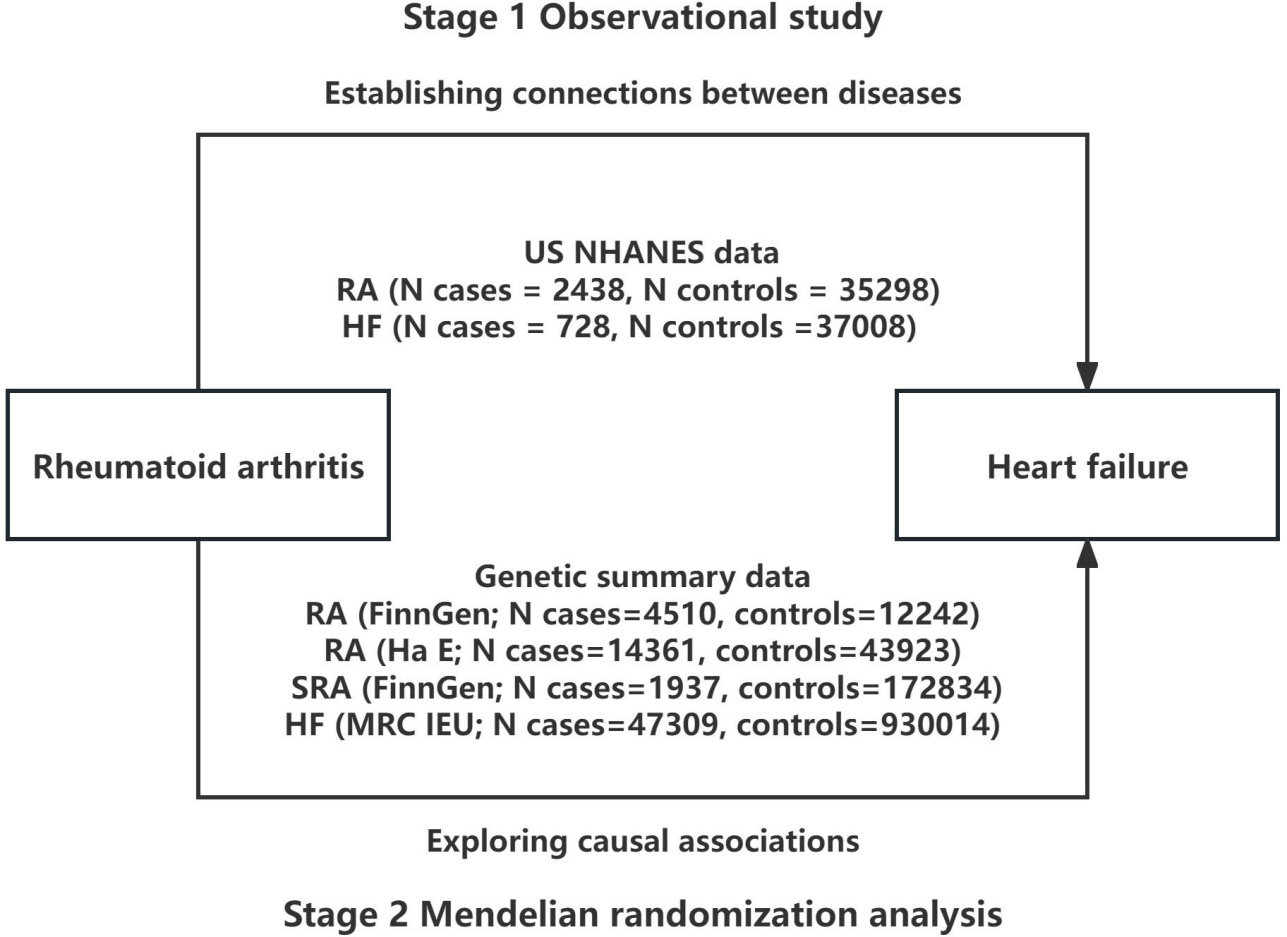
Overall study design based on observational analysis and Mendelian randomization. NHANES, National Health and Nutrition Examination Survey; RA, rheumatoid arthritis; SRA, seronegative rheumatoid arthritis; HF, heart failure.

As illustrated in **Figure 1**, this study comprises of two phases. In the initial phase, we aimed to investigate the association between RA and HF through a cross-sectional analysis utilizing data from the NHANES database. In the second phase, we obtained genetic instruments for RA from a meta-analysis of genome-wide association studies (GWAS) and used MR analysis to evaluate the causal relationship between genetically determined RA and HF. In cases where multiple independent datasets were accessible during MR analysis, we combined the findings through a meta-analytical approach.

### 2.2 Study population in NHANES

NHANES is a valuable resource for assessing the health and nutritional status of the US population. The survey uses a complex sampling design to ensure that the sample is representative of the non-institutionalized population, and collects data through interviews, physical examinations, and laboratory tests. The data collected from NHANES is used to inform public health policies and programs, as well as to monitor trends in health and nutrition over time. The NHANES survey was conducted in accordance with the ethical standards set by the National Center for Health Statistics Ethics Review Board. To learn more about the NHANES survey and access detailed information, please visit the official website of the Centers for Disease Control and Prevention at https://www.cdc.gov/nchs/index.htm. All participants provided written informed consent, and this study was based on a secondary analysis of publicly available data. The study was designed following the principles of the Guidelines for Strengthening the Reporting of Observational Studies in Epidemiology for reporting cross-sectional studies (14).

**Figure 2:**
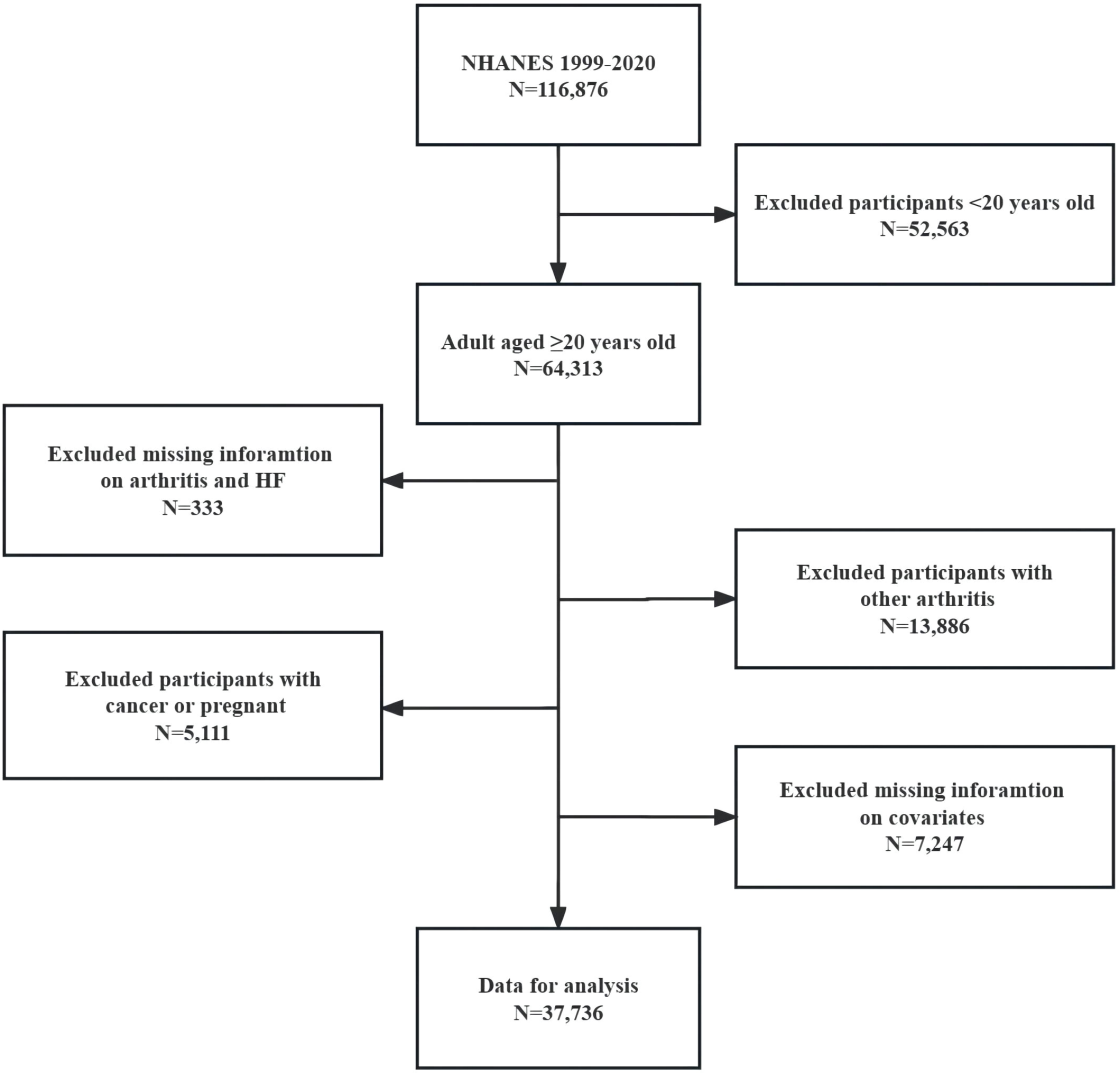
Flow chart of eligible NHANES participants included in this Study. NHANES, National Health and Nutrition Examination Survey; HF, heart failure.

We enrolled individuals who were 20 years of age or older and had complete information on all variables for NHANES cycles from 1999-2020. We excluded participants with other forms of arthritis, cancer, and those who were pregnant. Please refer to **Figure 2** for a detailed flow chart of our inclusion and exclusion criteria. The number and percentage of missing covariate data are shown in **Supplementary Table S1**.

### 2.3 RA and HF assessment in NHANES

In our observational study, we considered RA as an exposure variable and treated HF as an outcome variable. The study utilized a self-reported medical questionnaire to determine whether participants had RA or HF. Previous research has shown that self-reported disease in participants of large cross-sectional studies is a valid method of measurement (15, 16). The researchers used two questions to identify individuals with RA: “Have you ever been diagnosed with arthritis by a doctor or other health professional?”. If yes, “which type of arthritis were you diagnosed with?”. Because there is a lack of biomarker data for RA, its subtypes cannot be defined in detail in cross-sectional studies. They also used a question to identify individuals with HF: “Have you ever been informed by a physician or other healthcare provider that you have HF?”. Responding ‘yes’ to this question was indicative of HF.

### 2.4 Covariates used in NHANES

We made sure to include all relevant covariates in our study, based on previous research and data from NHANES (16). These covariates covered a wide range of demographics, lifestyle habits, medical history, laboratory tests, and physical measurements. Specifically, we included age, sex, race, education level, insurance coverage, body mass index (BMI), smoking, recreational activity, diabetes, hypertension, chronic renal failure, coronary heart disease, stroke, heart attack, angina, family history of heart disease, total cholesterol, triglycerides, and high-density lipoprotein. Socioeconomic factors were assessed and collected during the home interview, based on the original survey records. Behavioral factors were obtained through self-reports. A never smoker is an individual who has never smoked more than 100 cigarettes in their lifetime. Former smokers are defined as those who have smoked more than 100 cigarettes in their lifetime but have since quit smoking. Current smokers are individuals who have smoked at least 100 cigarettes in their lifetime and continue to smoke on some days or every day. BMI was measured at an mobile examination center using standardized protocols. Subject had self-reported medical history of angina pectoris, coronary heart disease, heart attack, or stroke, which was confirmed by a physician. The definition of hypertension is a diagnosis made by a healthcare professional, based on an average blood pressure reading of ≥130/80 mmHg or the use of hypertension medications. Diabetes was defined as a diagnosis made by a physician or other healthcare professional, glycated hemoglobin (%) greater than 6.5, random blood glucose (mmol/L) equal to or greater than 11.1, or use of diabetes medication or insulin. Chronic kidney disease is characterised by an estimated glomerular filtration rate <60 mL/min/1.73 m^2^ or a urine albumin–creatinine ratio of at least 30. A laboratory blood analyzer was used to collect the serum lipid profile, including total cholesterol, high-density lipoprotein, and triglyceride. For more detailed information about these covariates, please refer to **Supplementary Table S2**.

### 2.5 Genetic instruments for RA in MR

For RA, we utilized GWAS data from the FinnGen database (https://www.finngen.fi/en) and an independent study (17) as instrumental variables. Conversely, for seronegative RA (SRA), we relied on GWAS data solely from the FinnGen database. The GWAS analysis on RA sourced from the FinnGen database encompassed 4,510 cases and 12,242 controls. And data from an independent GWAS study comprised 14,361 cases and 43,923 controls. Conversely, the GWAS analysis on SRA involved 1,937 cases and 172,834 controls; The disease data utilized in this study offered clear and well-defined definitions and classifications. Specifically, the FinnGen Consortium employed International Classification of Diseases 9 and 10 codes to delineate RA and SRA. Furthermore, the inclusion and exclusion criteria for cases in the independent study were thoroughly elucidated in the original manuscript.all samples were from European populations, and all cases were included with reference to strict diagnostic criteria for confirmation.

We selected RA and SRA related single nucleotide polymorphisms (SNPs) at genome-wide significance (P < 5×10^–8^) as instrumental variables in secondary analysis to maximize specificity. Second, we request a number of bases between two SNPs (kb >10000) and further quality control was based on a minor allele frequency > 1% based on the population reference data from 1,000 Genomes Project (18). In addition, we manually checked all the identified SNPs by PhenoScanner GWAS database and excluded variants for the linkage disequilibrium. SNPs are not associated with confounders by the PhenoScanner GWAS database. The strength of the included SNPs was evaluated using the F statistic, with a threshold set at an F value greater than 10.

### 2.6 Genetic summary data of HF

Data on genetic variants connected with HF were taken from a GWAS, which collected 47,309 HF samples and 930,014 controls samples. SNPs and HF association data are available for download from the MRC IEU Open GWAS dataset. Participants of European ancestry from 26 cohorts (with a total of 29 distinct datasets) with either a case-control or population-based study design were included in the meta-analysis. Cases included participants with a clinical diagnosis of HF of any etiology without inclusion criteria based on left ventricular ejection fraction; controls were participants without HF (19).

### 2.7 Statistical analysis

In a cross-sectional study utilizing the NHANES database, baseline data was categorized based on the presence or absence of RA. Continuous variables were compared between groups using t-tests, while categorical variables were compared using chi-square tests. Multivariate logistic regression analysis was conducted to assess the association between RA and HF using odds ratios (ORs) and 95% confidence intervals (CIs). Model 1 was a crude model, model 2 adjusted for age, sex, and race, and model 3 adjusted for all covariates included in the study. Furthermore, we assessed the variance inflation factors (VIF) for each covariate and found that all VIF values were less than 5. This suggests that there was no significant multicollinearity among the covariates. At the same time, subgroup analyses within fully adjusted models were performed stratified by age (<60/≥60 years), sex (Male/Female), and race (Non-Hispanic white/Non-Hispanic black/Mexican American/Others), and multiplicative interactions were assessed using likelihood ratio tests. During data analysis, we followed NHANES analysis guidelines exactly (20), using sample weights, stratification and clustering variables to account for complex sampling designs that allow results to be generalized to the entire US population.

In this study, the main method of analysis for the MR analysis was inverse variance weighting (IVW). We merged the findings from an independent study, along with the dataset from FinnGen, using a fixed-effects model for meta-analysis (21). Additionally, we employed median weighted MR (22), mode-based MR, MR-Egger, and MR-PRESSO analyses (23) to assess the robustness and stability of our findings. The MR-Egger regression was employed to identify horizontal pleiotropy (24). Furthermore, We utilized Cochran’s Q test to evaluate potential heterogeneity among the SNPs. We applied MR-PRESSO method and a leave-one-out analysis to identify potential pleiotropic outliers, and MR-PRESSO conducts a global test of heterogeneity to identify potential horizontal pleiotropy and MR-Egger method was performed, which can adjust for bias from directional pleiotropic effects (25). The statistical analyses mentioned above were conducted using R software (Version 4.2.1) and we conducted the meta-analysis using STATA software (Version 12.0) to synthesize the collected data from the MR studies.

## 3 Results

### 3.1 Baseline characteristics of the sample population in observational study

**Table 1** presents the findings of the descriptive analysis conducted on the baseline characteristics of the sample. The observational study included a total of 37,736 participants who were 20 years of age or older and had complete variable data. Complex sample analysis indicated that this sample was representative of the 141,700,894 ambulatory population across the United States. Overall, participants had a mean age ± SD of 42.73 ± 0.16 years; 48.15% (95%CI 46.58 to 49.73) were women, and 64.13% (95%CI 60.53 to 67.73) were non- Hispanic white. The prevalence of HF in combination with RA reached 7.11% (95%CI 5.83 to 8.39). RA is more commonly found in the elderly, women, and individuals with lower levels of education and higher insurance coverage compared to their counterparts. Additionally, there are differences in the prevalence of RA among different races. In addition, We observed notable distinctions between individuals with and without RA in several factors, including smoking habits, participation in leisure activities, medical history, family history of CVD, and lipid profile (excluding high-density lipoprotein).

**Table 1:** General characteristics of included participants (n = 37,736) by the presence or absence of rheumatoid arthritis in the NHANES 1999–2020.

Characters	Overall (n=37,736)	Non-RA (n=35,298)	RA (n=2,438)	P-value
**Age, year**	42.73±0.16	42.05±0.16	56.53±0.38	< 0.0001
**Body mass index (kg/m^2^)**	28.40±0.07	28.31±0.07	30.32±0.21	< 0.0001
**Sex**				< 0.0001
Male	51.85(50.13-53.56)	52.26(51.71-52.81)	43.51(40.64-46.38)	
Female	48.15(46.58,49.73)	47.74(47.19-48.29)	56.49(53.62-59.36)	
**Race**				< 0.0001
Mexican American	9.90( 8.83-10.96)	10.03(8.88-11.17)	7.25(5.84- 8.66)	
Non-Hispanic Black	11.55(10.61-12.49)	11.26(10.21-12.31)	17.44(15.12-19.76)	
Non-Hispanic White	64.13(60.53-67.73)	64.15(62.17-66.14)	63.69(60.16,67.22)	
Other Hispanic	6.64( 5.81-748)	6.70(5.85-7.55)	5.45(4.14-6.76)	
Other race or multi-racial	7.78( 7.12-8.44)	7.86(7.18-8.53)	6.17(4.61-7.73)	
**Education**				< 0.0001
Less Than 9th Grade	5.43( 5.03-5.82)	5.23(4.83-5.62)	9.47(8.10-0.85)	
9-11th Grade	10.60( 9.97-11.24)	10.36( 9.76-10.97)	15.42(13.45-17.39)	
High School Grad	23.87(22.63-25.11)	23.56(22.73-24.40)	30.04(27.17-32.91)	
College degree	30.95(29.72-32.17)	30.91(30.08-31.75)	31.59(28.76-34.43)	
College or above	29.15(27.44-30.87)	29.93(28.51-31.35)	13.47(11.37-15.57)	
**Insurance coverage**	79.42(76.59-82.25)	79.00(78.03-79.97)	87.91(86.06-89.76)	< 0.0001
**Smoking status**				< 0.0001
Now	21.61(20.58-22.63)	21.40(20.62-22.19)	25.68(23.06-28.29)	
Former	21.66(20.57-22.74)	21.15(20.44-21.87)	31.80(28.95-34.66)	
Never	56.74(54.86-58.61)	57.44(56.47-58.42)	42.52(39.41-45.63)	
**Vigorous recreational activity**	33.98(32.45-35.51)	34.87(33.76-35.98)	16.04(13.66-18.42)	< 0.0001
**Moderate recreational activity**	50.27(48.31-52.24)	50.77(49.64-51.89)	40.35(37.64-43.06)	< 0.0001
**Family history of heart disease**	12.20(11.54-12.86)	11.67(11.19-12.15)	22.83(20.38-25.28)	< 0.0001
**Diabetes**	8.81( 8.44-9.19)	8.20( 7.85-8.55)	21.20(19.28-23.13)	< 0.0001
**Hypertension**	41.54(39.95-43.14)	40.24(39.37-41.11)	67.79(65.04-70.54)	< 0.0001
**Chronic renal failure**	10.63(10.15-11.12)	10.07( 9.63-10.51)	22.06(20.02-24.10)	< 0.0001
**Heart failure**	1.27( 1.13-1.42)	0.98(0.87-1.10)	7.11(5.83-8.39)	< 0.0001
**Coronary heart disease**	2.07( 1.85-2.29)	1.81(1.62-2.01)	7.15(5.78-8.52)	< 0.0001
**Angina**	1.38( 1.21-1.55)	1.13(0.98-1.28)	6.33(5.02-7.63)	< 0.0001
**Heart attack**	2.06( 1.85-2.26)	1.71(1.55-1.88)	8.97(7.46-10.48)	< 0.0001
**Stroke**	1.63( 1.47-1.79)	1.35(1.22-1.48)	7.25(5.84-8.66)	< 0.0001
Lipid Profile				
Total cholesterol (mg/dL)	194.13±0.37	194.00±0.38	196.75±1.24	0.028
High-density lipoprotein (mg/dL)	52.67±0.17	52.67±0.17	52.61±0.43	0.880
Triglycerides (mg/dL)	145.79±1.12	145.26±1.14	156.47±3.13	< 0.001

Values indicate the weighted mean±SD or weighted % (95% CI). P values are weighted. RA, rheumatoid arthritis; NHANES, National Health and Nutrition Examination Survey.

### 3.2 Association between RA and HF in observational study

**Table 2** shows the relationship between RA and HF, as determined by a multivariate logistic regression analysis. As the model was adjusted from crude to fully adjusted, it was found that individuals with RA had a greater likelihood of developing HF compared to those without RA. The ORs (95% CIs) for the prevalence of HF across the model 1 to model 3 were 7.71 (6.26-9.49), 3.47 (2.77-4.33) and 1.93 (1.47-2.54), respectively. **Table 3** displays the outcomes of our subgroup analyses, focusing on age, sex, and race. The likelihood ratio test for the interaction between age and sex in relation to RA did not yield statistically significant results. Therefore, we can conclude that the findings from the primary analysis remain consistent and reliable. However, when subgroup analysis based on race was conducted, it was found that Non-Hispanic Black have a lower risk of developing HF as a result of RA compared to Non-Hispanic White and Mexican American (P interaction = 0.012).

**Table 2:** Weighted multivariate logistic regression coefficients (ORs) and 95% CIs for the association between rheumatoid arthritis with heart failure: The United States, 1999–2020.

Rheumatoid arthritis	Case/participants	Model 1 OR (95%CI) P- value	Model 2 OR (95%CI) P- value	Model 3 OR (95%CI) P- value
No	519/35298	Reference	Reference	Reference
Yes	209/2438	7.71(6.26,9.49) P<0.0001	3.47(2.77,4.33) P<0.0001	1.93(1.47, 2.54) P<0.0001

Model 1 was a crude model; Model 2 was adjusted for the parameters in Model 1 plus age, sex, and race; and Model 3 was adjusted for the parameters in Model 2 plus education level, insurance coverage, body mass index, smoking, recreational activity, diabetes, hypertension, chronic renal failure, coronary heart disease, stroke, heart attack, angina, family history of heart disease, total cholesterol, triglycerides, and high-density lipoprotein.

**Table 3:** Subgroup analysis for the association between rheumatoid arthritis with heart failure.

Subgroup	Rheumatoid arthritis OR (95% CI), P- value		P for interaction
	No	Yes	
**Age**			0.380
≥60y	Reference	1.98(1.45,2.70) P<0.0001	
<60y	Reference	2.04(1.11, 3.74) P=0.021	
**Sex**			0.489
Male	Reference	1.92(1.25, 2.94) P=0.003	
Female	Reference	2.03(1.36, 3.03) P<0.001	
**Race**			0.012
Non-Hispanic White	Reference	2.43(1.68, 3.52) P<0.0001	
Non-Hispanic Black	Reference	1.65(1.03, 2.63) P=0.036	
Mexican American	Reference	2.48(1.10, 5.58) P=0.028	
Others	Reference	0.59(0.21, 1.67) P=0.594	

All presented covariates were adjusted (as Model 3) except the corresponding stratification variable.

### 3.3 Causal association between RA and HF in MR

**Figure 3:**
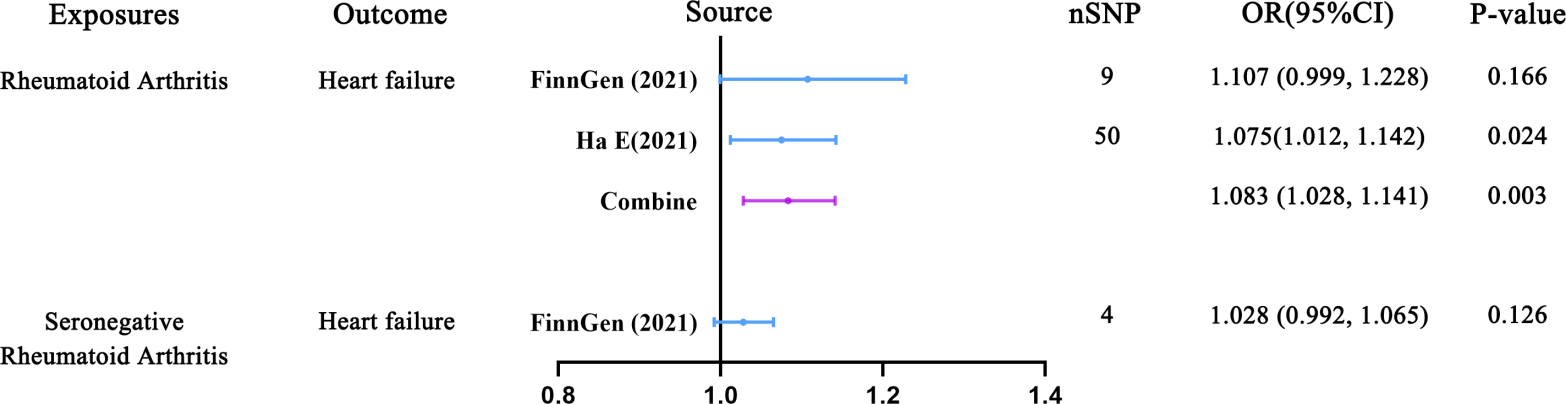
Predicting causal relationships between rheumatoid arthritis and heart failure using genetic analysis (Inverse variance weighted mendelian randomization).

In the MR analysis of RA and HF, 9 and 50 SNPs were utilized as IVs, respectively. Additionally, in the analysis of SRA as an exposure variable, 4 SNPs were also employed. As depicted in **Figure 3**; **Supplementary Figure 1**, in the main analysis employing a meta-analytical approach to combine the outcomes of the IVW method, we uncovered a significant causal relationship between genetically predicted RA and an increased risk of HF (OR = 1.083, 95% CI: 1.028-1.141; P = 0.003). Notably, the FinnGen Consortium dataset did not yield any significant causal association in the IVW model (OR = 1.107, 95% CI: 0.999-1.228; P = 0.166) (**Supplementary Figure 2**). Conversely, findings from GWAS utilizing independent datasets consistently indicated a significant causal association between RA and HF (OR = 1.075, 95% CI: 1.012-1.142; P = 0.024) (**Supplementary Figure 3**). Additionally, the outcomes of nearly all supplementary analytical techniques (including weighted MR, mode-based MR, MR-Egger, and MR-PRESSO analyses) corroborate the associations noted in GWAS studies using independent datasets, which is illustrated in **Supplementary Table 3**. However, as depicted in **Supplementary Figure 4**, this association was not deemed significant for SRA (OR = 1.028, 95% CI: 0.992-1.065; P = 0.126).

**Table 4** presents the results of the horizontal multiplicity and heterogeneity tests in the MR analysis conducted in this study. The MR-Egger regression analysis did not find any evidence of horizontal pleiotropy (All p > 0.05), indicating that the genetic variants used in the study do not have effects on the outcome through pathways other than the exposure of interest. Notably, while the Cochran Q test revealed heterogeneity among the included SNPs in the analysis, the meta-analysis did not yield significant heterogeneity(I^2^ = 0%). This suggests that the genetic instruments employed consistently influenced the exposure variables.In addition, the MR-PRESSO method and leave-one-out analysis did not detect any potential pleiotropic outliers (**Supplementary Tables 4**–**6**). Finally, The F statistics of all included SNPs were >10, indicating no weak-instrument bias.

**Table 4:** Assessing heterogeneity and pleiotropy in mendelian randomization analysis of rheumatoid arthritis and heart failure.

Exposure	Pleiotropy test		Heterogeneity test			Global test
	MR-Egger		IVW			MR-PRESSO
	Intercept	p-value	Q	Q-df	P-value	P-value
RA (FinnGen)	-0.005	0.097	70.033	49	0.026	0.763
RA (Ha E)	0.014	0.300	34.124	8	<0.001	0.785
SRA (FinnGen)	0.041	0.365	3.328	3	0.344	0.439

RA, rheumatoid arthritis; SRA, seronegative rheumatoid arthritis; IVW, inverse variance weighting.

## 4 Discussion

To the best of our knowledge, this study is the first to investigate the association between RA and HF using a large-scale cross-sectional study conducted at the national level, along with a genetically related MR study. In addition, our study also includes the analysis of a specific subtype of RA known as SRA. Our findings are based on two parts: in the cross-sectional study, we found that RA was linked to a nearly twofold higher prevalence of HF in the US population. In the MR study, we discovered a causal association between RA and HF.

Several epidemiological studies have shown a link between RA and HF. A previous study conducted over a 46-year period further supports this association (7), suggesting that RA is associated with a higher risk of developing HF. This increased risk cannot be solely attributed to traditional risk factors for CVD and ischemic heart disease. Furthermore, Danish population studies have also substantiated these claims (26). RA is a chronic and progressive disease. Certain factors have been found to be associated with an increased risk of HF, such as the use of corticosteroids and the presence of severe extra-articular manifestations. However, the use of methotrexate has been shown to decrease the risk of HF in these patients. It is important to note that this association is not a direct link between the two diseases, but rather can be influenced by factors such as the specific treatment approach, disease control, and various other factors (5, 27, 28). It is crucial for clinical practitioners to recognize that repeated flare-ups of RA symptoms can lead to a higher likelihood of rehospitalization in patients with HF resulting from the disease (29). This association may indicate a poorer prognosis (9, 30). There are subtle differences in the signs of HF between patients with RA and those without. Patients with RA may experience atypical symptoms and exhibit fewer signs of HF. Additionally, these patients often have a poorer prognosis, with features such as preserved ejection fractions, and they undergo cardiac ultrasonography less frequently (12, 31).

RA is a condition that causes immune disorders and a systemic inflammatory response. This can lead to the development of diastolic dysfunction HF due to myocardial remodeling, fibrosis, and impaired left ventricular diastolic function (32, 33). A proteomic study has also supported this perspective, revealing that HF resulting from RA is linked to increased expression of biomarkers associated with inflammation, such as tumor necrosis factor (TNF), fibrosis, and activation of the renin-angiotensin-aldosterone system (34). In RA, TNF plays a crucial role in promoting inflammation, along with other pro-inflammatory cytokines. It has been observed that increased levels of TNF are associated with disease activity (35). Consequently, the overexpression of TNF in RA may directly contribute to the development of HF, and this mechanism has been validated through animal experiments (36, 37). Cardiac biopsies from patients with inflammatory rheumatic diseases revealed a notable increase in the expression of adhesion molecules, pro-inflammatory cytokines, and human leukocyte antigens on both cardiomyocytes and endothelial cells (38). At the same time, research has demonstrated that elevated levels of C-reactive protein are linked to an increased risk of HF in individuals with RA. Conversely, the use of the anti-inflammatory medication methotrexate has been associated with a reduced risk of HF (5). In addition, there are theories suggesting that immune complexes that are highly specific to RA, such as anti-citrullinated protein antibodies, could potentially initiate autoimmune reactions in the heart, resulting in localized inflammation and restructuring of the myocardium (39, 40).

The association between RA and CVD has not only been demonstrated in epidemiological studies, but genetic susceptibility analysis further strengthens this association and indicates a causal relationship between the two conditions. A genetic study that examined the relationship between RA and cardiovascular and cerebrovascular diseases found positive associations with six outcomes: age angina, age heart attack, hypertension, abnormalities of heartbeat, stroke and coronary heart disease (41). This suggests that there are shared genetic mechanisms between RA and these cardiovascular and cerebrovascular conditions. Furthermore, additional studies have investigated the potential link between RA and other CVD. The findings indicate a notable connection with ischemic heart disease and myocardial infarction, rather than atrial fibrillation and arrhythmias (42). Similar to our study, a study has also conducted MR analysis on the relationship between RA and HF, focusing on the biomarker NT-proBNP. The findings of that study are consistent with the results of our study (43). However, this study did not take into account SRA, and the clinical significance of the reported lower OR value remains to be elucidated. In order to address these limitations, our study addresses this gap by combining real-world national cross-sectional studies.

SRA may present with atypical manifestations, making it challenging to diagnose clinically and easily overlooked in patient management (44, 45). Unfortunately, there has been a lack of comprehensive research on the relationship between SRA and HF, both in clinical studies and fundamental research. A case report highlighting severe cardiac tamponade and right HF in a patient with RA, who remained seronegative, emphasizes the need for rheumatologists to be aware of this condition (46). A large cohort study has provided confirmation that individuals with SRA do not face an increased risk of HF. However, it revealed that patients who tested positive for RA antibodies had a 36% higher risk of developing HF (11). Our genetic analysis unequivocally demonstrates the absence of a causal relationship between SRA and HF. Nevertheless, additional studies are warranted to corroborate our results and delve deeper into the underlying mechanisms at play.

Our research methodology is a unique strength of our study, as we utilized both cross-sectional data and MR to examine the relationship between RA and HF. By utilizing national-level cross-sectional studies, we were able to derive ORs that are applicable to real-world situations. Additionally, the use of MR analysis helped address the limitation of the former approach by providing a means to infer causality. This approach also allowed us to overcome the limitations of previous studies and provide more clinically relevant findings (43). Furthermore, we expanded our analysis to include SRA as an exposure variable, which enhances the comprehensiveness and depth of our study conclusions.

However, it is important to acknowledge the limitations of this study. Firstly, it’s worth noting that self-reported data from cross-sectional studies utilizing RA and HF lack details, such as disease duration and subtype. Due to the unavailability of data on biomarkers for RA, further investigation into these aspects is not feasible, despite the significance of RA biomarkers in diagnosis, disease prognosis, and management. Moreover, self-reported clinical data may be susceptible to various biases, including information bias and recall bias, among other potential limitations. Nevertheless, the currently published NHANES-based studies have demonstrated the significant epidemiological research value of self-reported diseases. In the study published in the Lancet sub-journal, which also utilized the NHANES database as the data source, the diagnosis of RA, similar to our study, was also obtained through self-report (47). In the US National Prevalence and Temporal Trends Study of Anemia in HF Patients, participants were also diagnosed with HF through self-report (48). These cross-sectional studies encompass a large number of individuals on a national scale, making it impractical for researchers to determine disease status using strict clinical diagnostic criteria, which would require substantial time and effort. Moreover, additional confounding variables, such as undisclosed patient treatment information, may not have been taken into account, potentially influencing our results. Our findings indicate that genetically predicted associations were not influenced by the use of these drugs, and rheumatoid arthritis demonstrated a causal relationship with heart failure at the genetic level. In MR studies, we cannot completely eliminate the possibility of horizontal pleiotropy during analysis, which can introduce bias. However, it is worth noting that the statistical analysis in this study, specifically MR-Egger, did not indicate any signs of horizontal pleiotropy. Furthermore, it is important to mention that our study primarily focused on European populations, and caution should be exercised when generalizing the findings to other populations. At the same time, we noted relatively small ORs in our MR results, consistent with previous studies (43). It is important to recognize that these findings do not automatically imply that exposed populations are at low risk, as the variability in exposure factors may surpass that explained by genetic variation alone. Additionally, the primary objective of MR is not to determine the precise magnitude of genetic effects but rather to evaluate the causal impact of environmental exposure on outcomes. Fortunately, our cross-sectional surveys effectively captured the true effect size between the two diseases.

## 5 Conclusion

In summary, our study indicates that RA correlates with an elevated prevalence of HF within the US population. Furthermore, genetic evidence derived from European populations underscores a causal link between RA and the risk of HF. However this association was not significant in SRA. Therefore, additional research is imperative to corroborate these results and delve into the underlying mechanisms driving this association.

## Data availability statement

The datasets presented in this study can be found in online repositories. The names of the repository/repositories and accession number(s) can be found in the article/**Supplementary Material**.

## Ethics statement

The studies involving humans were approved by Ethics Review Committee of the National Center for Health Statistics. The studies were conducted in accordance with the local legislation and institutional requirements. The participants provided their written informed consent to participate in this study.

## Author contributions

KK: Conceptualization, Formal analysis, Methodology, Writing – original draft, Writing – review & editing. DD: Methodology, Formal analysis, Visualization, Writing – original draft, Writing – review & editing. XZ: Conceptualization, Formal analysis, Methodology, Writing – original draft, Writing – review & editing. HL: Data curation, Writing – original draft. LK: Data curation, Writing – original draft. JH: Data curation, Writing – original draft. XC: Data curation, Writing – original draft. TL: Conceptualization, Resources, Supervision, Writing – review & editing. FK: Conceptualization, Resources, Writing – review & editing. XL: Conceptualization, Resources, Supervision, Writing – review & editing.
