# Supplementary material for: Rheumatoid arthritis increases the risk of heart failure: results from the cross-sectional study in the US population and mendelian randomization analysis in the European population
Source: Front Immunol. 2024 May 28;15:1377432. doi: 10.3389/fimmu.2024.1377432 (PMC11165030; doi:10.3389/fimmu.2024.1377432)
Supplement: Supplementary file 1 [file DataSheet_1.docx]

Supplementary Material

Rheumatoid arthritis increases the risk of heart failure: results from the cross-sectional study in the US population and mendelian randomization analysis in the European population

**Supplementary Table 1** The numbers and percentages of missing covariate data

**Supplementary Table 2** Definition and Details of Covariates

**Supplementary Table 3** Details on the five methods employed in this MR study.

**Supplementary Table 4**  Leave-one-out sensitivity analysis for heart failure using SNP associated rheumatoid arthritis (FinnGen)

**Supplementary Table 5** Leave-one-out sensitivity analysis for heart failure using SNP associated rheumatoid arthritis (Ha E)

**Supplementary Table 6** Leave-one-out sensitivity analysis for heart failure using SNP associated Seronegative rheumatoid arthritis (FinnGen)

**Supplementary Figure 1** Forest plot of the effect of rheumatoid arthritis on heart failure (Meta-analysis)

**Supplementary Figure 2** (A) Funnel plot; (B) Scatter plot; (C) Forest plot; (D) Sensitivity analysis of the effect of rheumatoid arthritis (FinnGen) on heart failure (Mendelian randomization)

**Supplementary Figure 3** (A) Funnel plot; (B) Scatter plot; (C) Forest plot; (D) Sensitivity analysis of the effect of rheumatoid arthritis (Ha E) on heart failure (Mendelian randomization).

**Supplementary Figure 4** (A) Funnel plot; (B) Scatter plot; (C) Forest plot; (D) Sensitivity analysis of the effect of Seronegative rheumatoid arthritis (FinnGen) on heart failure (Mendelian randomization)

**Supplementary Table 1** The numbers and percentages of missing covariate data

| **Covariate** | **Numbers** | **Percentages (%)** |
| --- | --- | --- |
| Education | 75 | 0.17 |
| Insurance coverage | 247 | 0.55 |
| Smoking status | 41 | 0.09 |
| Vigorous recreational activity | 13 | 0.03 |
| Moderate recreational activity | 15 | 0.03 |
| Family history of heart disease | 1112 | 2.47 |
| Diabetes | 2 | 0.004 |
| Hypertension | 48 | 0.11 |
| Chronic renal failure | 5121 | 11.38 |
| Coronary heart disease | 100 | 0.22 |
| Angina | 79 | 0.18 |
| Heart attack | 46 | 0.10 |
| Stroke | 32 | 0.07 |
| Total cholesterol | 4931 | 10.96 |
| High-density lipoprotein | 4932 | 10.96 |
| Triglycerides | 5065 | 11.26 |

**Supplementary Table 2** Definition and Details of Covariates

| **Covariate** | **Definition and NHANES codon** | **Grouping for adjustment** |
| --- | --- | --- |
| Age | Age at Screening (RIDAGEYR) | Continuous Variables |
| Sex | Gender (RIAGENDR) | Male; Female |
| Race | Race/Ethnicity (RIDRETH1) | Non-Hispanic White; Non-Hispanic Black; Mexican American; Other Hispanic; Other race/ethnicity |
| Education level | Education Level - Adults 20+ (DMDEDUC2) | Less Than 9th Grade; 9-11th Grade; High School Grad; College degree; College or above |
| Insurance coverage | A ratio of family income to poverty threshold (INDFMPIR) | Yes; No |
| Body mass index | Body Mass Index (kg/m^2^) (BMXBMI) | Continuous Variables |
| Smoking status | Never smokers: smoking less than 100 cigarettes in their life. Former smokers: smoked more than 100 cigarettes in their life and had quit smoking. Current smokers smoked more than 100 cigarettes in their life and smoked some days or every day. (SMQ020; SMQ040) | Current smokers; Former smokers; Never smokers |
| Vigorous recreational activity | over the past 30 days, do any vigorous activities for at least 10 minutes (PAQ650; PAQ665) | Yes; No |
| Moderate recreational activity | over the past 30 days, do any Moderate activities for at least 10 minutes (PAD200; PAD320) | Yes; No |
| Family history of heart disease | Including living and deceased, were any of your close biological that is, blood relatives ever told by a health professional that they had a heart attack or angina before the age of 50? (MCQ250G;MCQ300A) | Yes; No |
| Hypertension | Diagnosis by a doctor or other health professional, average blood pressure ≥130/80 mmHg or use of hypertension medication. (BPQ020; BPXSY1; BPXDI1; BPXSY2; BPXDI2; BPXSY3; BPXDI3; BPQ040A) | Yes; No |
| Diabetes | Diagnosis by a doctor or other health professional, glycohemoglobin (%) >6.5, random blood glucose (mmol/l) ≥11.1, or use of diabetes medication or insulin. (DIQ010; DIQ050; DIQ070; LBXGH; LBDSGLSI) | Yes; No |
| Chronic renal failure | Estimated glomerular filtration  rate <60 mL/min/1.73 m^2^ or a urine albumin–creati-  nine ratio of at least 30. (URXUCR; URDACT) | Yes; No |
| Coronary heart disease | Has a doctor or other health professional ever told you had coronary heart disease? (MCQ160C) | Yes; No |
| Angina | Has a doctor or other health professional ever told you had Angina? (MCQ160D) | Yes; No |
| Heart attack | Has a doctor or other health professional ever told you had Heart attack? (MCQ160E) | Yes; No |
| Stroke | Has a doctor or other health professional ever told you had Stroke? (MCQ160F) | Yes; No |
| Total cholesterol (mg/dL) | Laboratory (LBXTC) | Continuous Variables |
| High-density lipoprotein (mg/dL) | Laboratory (LBDHDL; LBXHDD; LBDHDD) | Continuous Variables |
| Triglycerides (mg/dL) | Laboratory (LBXSTR) | Continuous Variables |

**Supplementary Table 3** Details on the five methods employed in this MR study.

| **Exposures** | **Outcome** | **nSNP** | **OR** | **95%CI** | **P value** |
| --- | --- | --- | --- | --- | --- |
| **Rheumatoid Arthritis** | **Heart Failure** |  |  |  |  |
|  | **FinnGen** |  |  |  |  |
|  | MR Egger | 9 | 0.988 | 0.878, 1.112 | 0.851 |
|  | Weighted median | 9 | 1.040 | 0.997, 1.084 | 0.067 |
|  | Inverse variance weighted | 9 | 1.046 | 0.981, 1.116 | 0.166 |
|  | Simple mode | 9 | 1.050 | 0.996, 1.108 | 0.109 |
|  | Weighted mode | 9 | 1.043 | 1.004, 1.085 | 0.063 |
|  | **Ha E** |  |  |  |  |
|  | MR Egger | 50 | 1.054 | 1.014, 1.096 | 0.011 |
|  | Weighted median | 50 | 1.043 | 1.015, 1.073 | 0.003 |
|  | Inverse variance weighted | 50 | 1.024 | 1.003, 1.045 | 0.024 |
|  | Simple mode | 50 | 1.029 | 0.976, 1.084 | 0.292 |
|  | Weighted mode | 50 | 1.038 | 1.007, 1.071 | 0.021 |
| **Seronegative Rheumatoid Arthritis** | **Heart Failure** |  |  |  |  |
|  | **FinnGen** |  |  |  |  |
|  | MR Egger | 4 | 0.923 | 0.768, 1.110 | 0.486 |
|  | Weighted median | 4 | 1.020 | 0.978, 1.063 | 0.358 |
|  | Inverse variance weighted | 4 | 1.028 | 0.992, 1.065 | 0.126 |
|  | Simple mode | 4 | 0.999 | 0.944, 1.059 | 0.987 |
|  | Weighted mode | 4 | 1.022 | 0.974, 1.072 | 0.434 |

**Supplementary Table 4** Leave-one-out sensitivity analysis for heart failure using SNP associated rheumatoid arthritis (FinnGen)

| SNP | BETA | SE | P-value |
| --- | --- | --- | --- |
| rs114484678 | -0.170087065 | 0.064365672 | 0.008229353 |
| rs11571293 | 0.054961832 | 0.061068702 | 0.368120251 |
| rs17805996 | -0.045180723 | 0.084939759 | 0.594784941 |
| rs3129294 | 0.042320819 | 0.058020478 | 0.465749823 |
| rs62395272 | 0.02968251 | 0.026300958 | 0.259078669 |
| rs6679677 | 0.068938807 | 0.032274722 | 0.032679414 |
| rs7137828 | 0.375796178 | 0.072793449 | 2.44E-07 |
| rs7574865 | 0.018853695 | 0.071644042 | 0.792428882 |
| rs7731626 | 0.067746686 | 0.063328424 | 0.284724001 |
| rs114484678 | -0.170087065 | 0.064365672 | 0.008229353 |
| rs11571293 | 0.054961832 | 0.061068702 | 0.368120251 |
| rs17805996 | -0.045180723 | 0.084939759 | 0.594784941 |
| All | 0.045327018 | 0.032722332 | 0.165990916 |

**Supplementary Table 5** Leave-one-out sensitivity analysis for heart failure using SNP associated rheumatoid arthritis (Ha E)

| SNP | BETA | SE | P-value |
| --- | --- | --- | --- |
| rs10175798 | 0.105595763 | 0.093991833 | 0.261243542 |
| rs11217044 | -0.179350978 | 0.074029978 | 0.015406474 |
| rs11574914 | -0.092650601 | 0.074120481 | 0.211299547 |
| rs11889341 | 0.042738957 | 0.072503587 | 0.55554356 |
| rs11933540 | -0.038057762 | 0.063908317 | 0.551505545 |
| rs12126142 | 0.148713865 | 0.095944429 | 0.121141516 |
| rs12466919 | -0.025589214 | 0.071473321 | 0.720324836 |
| rs13142500 | -0.036051053 | 0.130419984 | 0.782223374 |
| rs1571878 | 0.010608462 | 0.052379279 | 0.839501121 |
| rs1858036 | 0.090885828 | 0.072355708 | 0.2090806 |
| rs187786174 | -0.077230679 | 0.108122951 | 0.475050524 |
| rs1893592 | -0.026830203 | 0.083365274 | 0.747574606 |
| rs1953126 | 0.114878907 | 0.095152226 | 0.227310058 |
| rs2105325 | 0.066438246 | 0.085420602 | 0.436700031 |
| rs212389 | -0.018885702 | 0.084985657 | 0.824140896 |
| rs2233424 | -0.135864862 | 0.086105438 | 0.11459101 |
| rs2235924 | -0.082705356 | 0.090127631 | 0.358803672 |
| rs2301888 | 0.017992224 | 0.068839814 | 0.793812097 |
| rs2317230 | 0.068866049 | 0.103948753 | 0.507650835 |
| rs2561477 | 0.154710392 | 0.103140261 | 0.133614403 |
| rs2736337 | 0.08162413 | 0.089217073 | 0.360247481 |
| rs28411352 | -0.140299482 | 0.078532414 | 0.074015611 |
| rs2858329 | 0.005153634 | 0.029353309 | 0.860629794 |
| rs3087243 | 0.056727607 | 0.057445678 | 0.323397583 |
| rs3778753 | 0.011389414 | 0.074980306 | 0.879266807 |
| rs3784099 | -0.027568452 | 0.092248281 | 0.76505406 |
| rs3806624 | -0.038377772 | 0.095944429 | 0.689156517 |
| rs4239702 | -0.0643589 | 0.075514442 | 0.394062739 |
| rs4409785 | -0.042710301 | 0.099657368 | 0.668235142 |
| rs5019428 | 0.071944366 | 0.133445195 | 0.589796857 |
| rs6679677 | 0.04500048 | 0.021067641 | 0.032679414 |
| rs6712515 | 0.041203526 | 0.07474128 | 0.581440341 |
| rs6930468 | -0.174953637 | 0.088006981 | 0.04681702 |
| rs706778 | 0.010443537 | 0.091671047 | 0.909298 |
| rs71508903 | -0.014012382 | 0.064966498 | 0.829232301 |
| rs73013527 | -0.044533653 | 0.084826006 | 0.599583191 |
| rs73081554 | 0.040479959 | 0.095460203 | 0.671528887 |
| rs76153210 | 0.161537887 | 0.161537887 | 0.317310508 |
| rs773125 | 0.167096592 | 0.095152226 | 0.079071752 |
| rs7731626 | 0.049374765 | 0.046154672 | 0.284724001 |
| rs7752903 | 0.027701337 | 0.072449651 | 0.702199591 |
| rs7754520 | 0.105836138 | 0.030950036 | 0.000627178 |
| rs8026898 | 0.059291201 | 0.059291201 | 0.317310508 |
| rs8032939 | 0.149312647 | 0.078088798 | 0.055864919 |
| rs8083786 | 0.128292381 | 0.080573874 | 0.111332359 |
| rs9277411 | 0.041712724 | 0.030936937 | 0.177557216 |
| rs9348832 | -0.00431979 | 0.078934347 | 0.956356462 |
| rs947474 | -0.005749329 | 0.09965504 | 0.953993721 |
| rs9603616 | 0.046506772 | 0.078776777 | 0.554948353 |
| rs9747973 | -0.296891021 | 0.083765681 | 0.000393651 |
| All | 0.023761672 | 0.010558436 | 0.02441775 |

**Supplementary Table 6** Leave-one-out sensitivity analysis for heart failure using SNP associated seronegative rheumatoid arthritis (FinnGen)

| SNP | BETA | SE | P-value |
| --- | --- | --- | --- |
| rs10947233 | -0.006185931 | 0.038882998 | 0.87359725 |
| rs6679677 | 0.085549503 | 0.040051266 | 0.032679414 |
| rs9274507 | 0.028993011 | 0.025110018 | 0.248238217 |
| rs9296004 | -0.002768379 | 0.043986466 | 0.949816621 |
| All | 0.027686328 | 0.018096391 | 0.126032462 |

**Supplementary Figure 1** Forest plot of the effect of rheumatoid arthritis on heart failure (Meta-analysis).

**
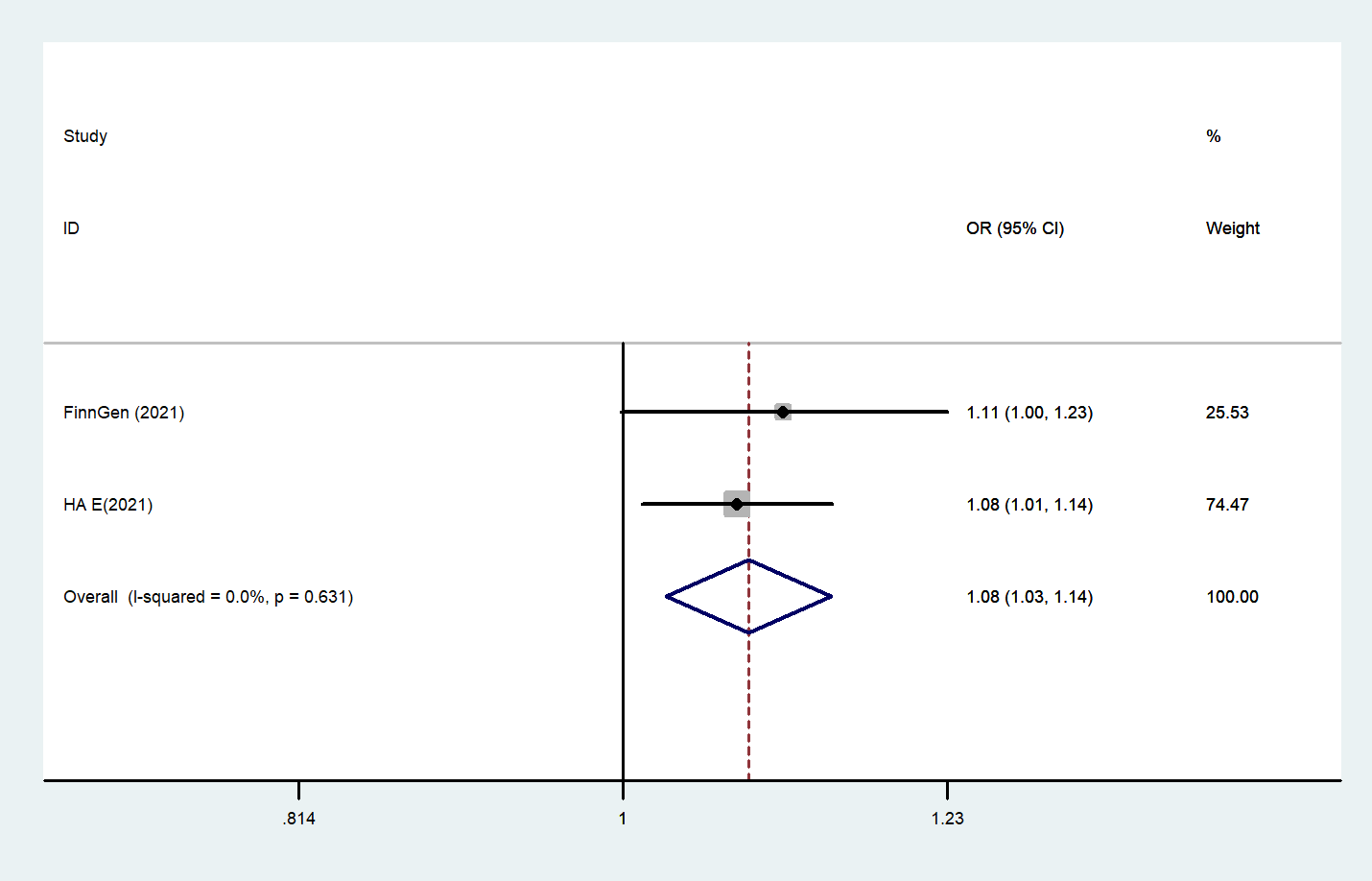
**

**Supplementary Figure 2** (A) Funnel plot; (B) Scatter plot; (C) Forest plot; (D) Sensitivity analysis of the effect of rheumatoid arthritis (FinnGen) on heart failure (Mendelian randomization).

**
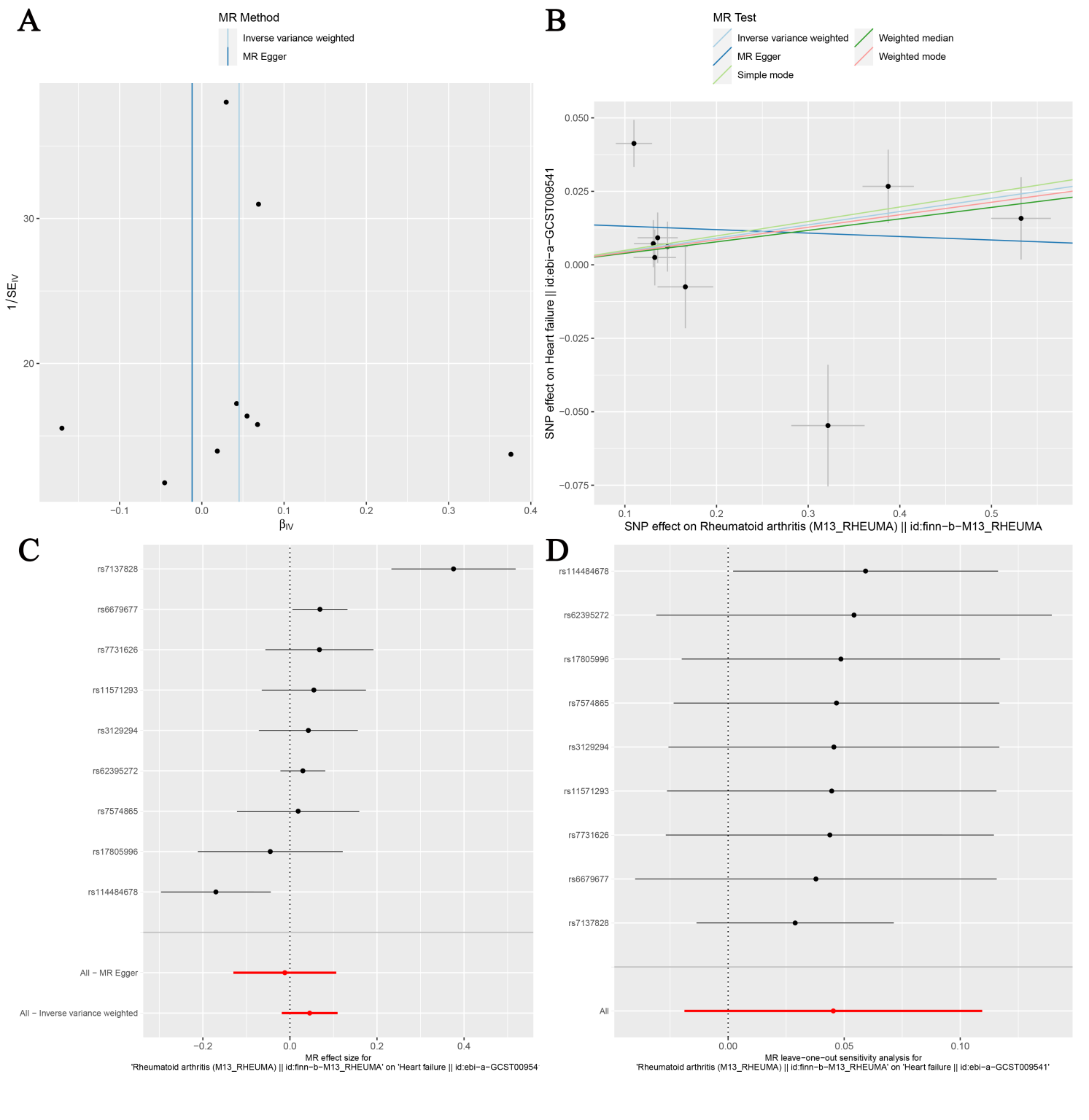
**

**Supplementary Figure 3**  (A) Funnel plot; (B) Scatter plot; (C) Forest plot; (D) Sensitivity analysis of the effect of rheumatoid arthritis (Ha E) on heart failure (Mendelian randomization).

**
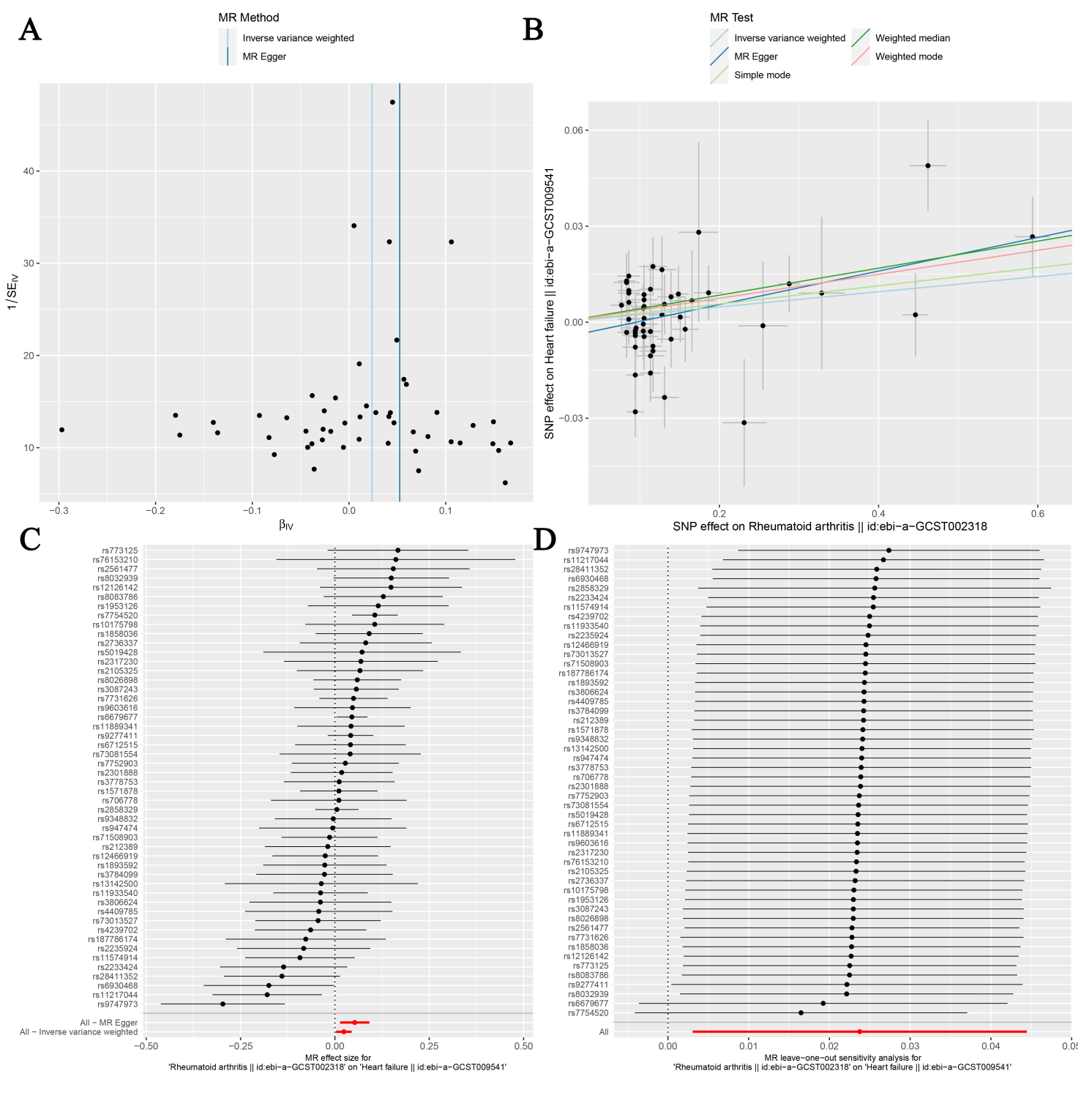
**

**Supplementary Figure 4**  (A) Funnel plot; (B) Scatter plot; (C) Forest plot; (D) Sensitivity analysis of the effect of seronegative rheumatoid arthritis (FinnGen) on heart failure (Mendelian randomization).

**
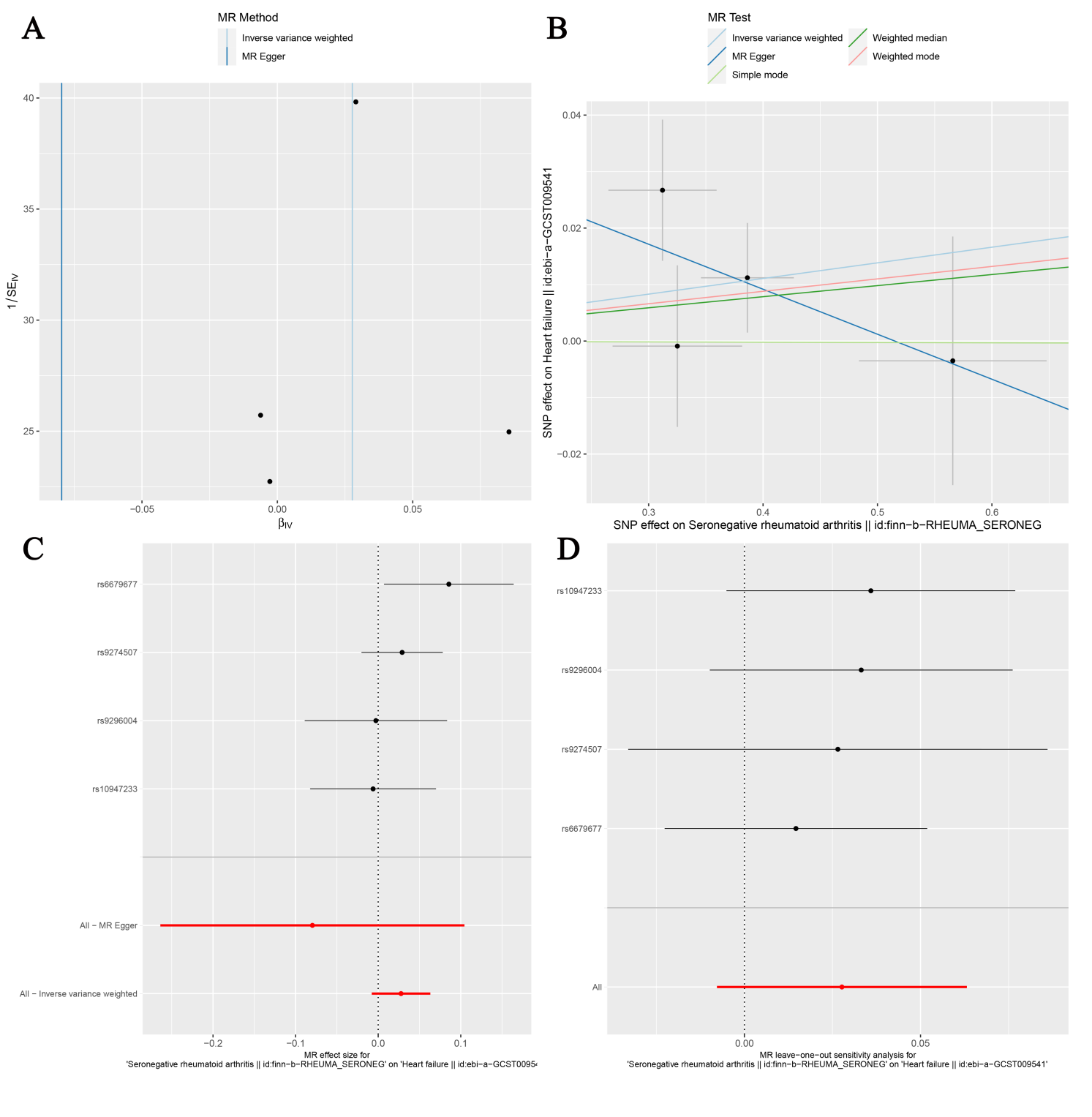
**
